# Effects of water volume of drip irrigation on soil bacterial communities and its association with soil properties in jujube cultivation

**DOI:** 10.3389/fmicb.2023.1321993

**Published:** 2024-01-19

**Authors:** Zhaoyang Li, Yuhui Yang, Jiangfan Liu, Wenge Jiang, Yang Gao

**Affiliations:** ^1^College of Water Hydraulic and Architectural Engineering, Tarim University, Alar, China; ^2^Laboratory of Modern Agricultural Engineering, Tarim University, Alar, China; ^3^Key Laboratory of Northwest Oasis Water-Saving Agriculture, Ministry of Agriculture and Rural Affairs, Shihezi, China; ^4^Institute of Farmland Irrigation, Chinese Academy of Agricultural Sciences, Xinxiang, Henan, China

**Keywords:** available nutrient levels, bacterial community assembly, drip irrigation, optimal water amount, jujube yields

## Abstract

**Introduction:**

Jujube is one of an important crop in Xinjiang, China, a area suffered by water scarcity and DI has been proven as a suitable mode for jujube cultivation. Soil bacterial community play a vital role in biogeochemical cycles to support the crop growth, and water content is considered as one of the important factors for them. However, limited research has explored the optimum irrigation strategies, such as water volume of DI, to maximize the benefits of jujube cultivation by regulating the soil bacterial communities.

**Methods:**

Therefore, in this study, we conducted DI experiments on jujube fields in Xinjiang with three different water volume levels, and measured the soil properties and bacterial communities of the flowering and fruit setting (FFS) and end of growth (EG) stages.

**Results and discussion:**

Significant lower jujube yield and soil available nutrients were observed in samples with low water amount. In addition, we discovered significant effects of the water amount of DI and jujube growth stages on soil bacterial communities. Based on the compare of samples among different growth stages and water amounts some growth stage related bacterial genera (*Mycobacterium, Bradyrhizobium*, and *Bacillus*) and water amount-related bacterial phyla (Chloroflexi, Nitrospirota, and Myxococcota) were recognized. Moreover, according to the results of null model, soil bacterial communities were governed by stochastic and deterministic processes under middle and low water volumes of DI, respectively. Finally, we deduced that middle water amount (600 mm) could be the optimal condition of DI for jujube cultivation because the higher jujube yield, deterministic assembly, and stronger correlations between soil properties and bacterial community under this condition. Our findings provide guidance for promoting the application of DI in jujube cultivation, and further research is needed to investigate the underlying mechanisms of soil bacterial community to promote the jujube yield.

## 1 Introduction

Jujube is an ancient, native species in China and has been contiunually cultivated for more than 4,000 years (Sheng and Shen, 2011). In 2018, China produced 8.78 million tons of jujube, which comprised 98% of the total worldwide yield (Wang et al., 2020). Currently, the Xinjiang Uygur Autonomous Region is the largest jujube-producing area in China, in which, most crops have been planted with DI technology due to the shortage of water resources (Wang et al., 2019). However, most jujube trees were irrigated by traditional flood irrigation methods, which have low water use efficiency (Lu et al., 2014). At the same time, long-term flood irrigation in arid areas can easily lead to soil compaction and secondary salinization, which results in a reduction in crop yields and soil quality (Yang et al., 2020; Zhang et al., 2020b). In recent years, Xinjiang has vigorously promoted the application of DI technology in jujube orchards. Many scholars have conducted extensive research on the water and fertilizer utilization efficiency of jujube trees after the irrigation method changed from flood to drip. One previous study reported that DI aided during the cell expansion period of jujube fruit growth to conserve water while ensuring that the fruit receives adequate moisture (Wang et al., 2021). Another study revealed that DI could reconstruct the root distribution of jujube trees and improve water use efficiency (Li et al., 2021). Moreover, one study has been performed on appropriate water and fertilizer scheduling that can significantly improve the yield of jujube trees after flood irrigation changed to DI (Wang et al., 2018). DI has been found to be an efficient and suitable irrigation method for jujube cultivation (Yang et al., 2023).

In addition to the direct effects on crop growth by regulating the moisture of soils, DI also could influence the crop yield via the variation of other abiotic and biotic factors (Hartmann and Six, 2023). The available nutrient levels (ANL) for crops in soils are directly related to crop growth (Timsina, 2018; Moon and Ali, 2022). DI technique has proven to improve the ANL to the roots of plants and enhance crop yields (Shedeed et al., 2009; Tiwari et al., 2014). More importantly, the soil bacterial community plays a vital role in the biogeochemical cycles of critical elements, which act as producers to support the soil productivity of agriculture systems (Zhou et al., 2019). Interactions between the soil bacterial communities and crop growth have been extensively investigated in agricultural ecosystems covering diverse crop species and environmental conditions (Chen et al., 2019; Guo et al., 2020; Li et al., 2022). Water content in agricultural soils is one of the important influencing factors for bacterial communities living within them (Gupta et al., 2022). In addition, the soil bacterial community will also change by other soil properties related to soil moisture, such as pH, ANL, and soil enzyme activities (SEAs; Zhao et al., 2018; Jiao and Lu, 2020). Some investigations have reported the change in soil bacterial communities in agricultural ecosystems under different irrigation strategies (Zhou et al., 2019; Tian et al., 2022).

Although many scholars have studied the effects of different drip irrigation water and fertilizer scheduling on the physiological growth and yield of jujube trees after the irrigation method changed from flood to drip, however, the effects of water volume in DI on soil properties and bacterial communities as well as their underlying associations in jujube cultivation are not fully understood. To explore the ideal water volume for DI in jujube cultivation, we established a DI assay of jujube fields in Xinjiang with three different water volume levels. Soil samples were collected from these fields at both the flowering and fruit setting (FFS) and end of growth (EG) stages to measure soil properties and bacterial communities. The final jujube yield of different treatments was also recorded. The objectives of this study are: (i) to determine the effects of water volume in DI on soil properties and jujube yields; (ii) to ascertain variations in soil bacterial communities as a function of water volume in DI; and (iii) to determine the relationship between soil properties and bacterial communities under different water volumes of DI. Based on the results of this study, we could clarify the underlying mechanisms of variations of soil bacterial communities and guide the operation condition of DI technology during jujube cultivation.

## 2 Materials and methods

### 2.1 Experimental site and field treatments

The experiment was conducted in the Experimental Station (81°13′E, 40°34′N; altitude of 1,015 m) of the 10th Regiment of the First Division of the Xinjiang Production and Construction Corps of China in 2019. The site was located on the northern edge of the Taklimakan Desert, with a typical continental arid desert climate that is characterized by hot summers with low rainfall, cold winters with little snow, and strong surface evaporation. According to data collected at the Aksu Meteorological Station, over the past 30 years, the average annual rainfall and evapotranspiration are 50 and 2,200 mm, respectively. The annual sunshine duration in this region was 2,556–2,992 h, the annual mean temperature was 8.4°C–11.4°C, and the annual frost–free season lasts for 180–221 d. The rainfall during the jujube growth period (April–October) was 61.2 mm. The depth of the groundwater table was more than 3.5 m.

Jujube trees (*Zizyphus jujube* Mill) selected in this study were planted in 2007, grafted in 2008, and cultivated for 12 years. The trees were densely planted with a row spacing of 2 m and plant spacing of 0.8 m, Planting density reached 6,253 plants ha^−1^. From 2007 to 2017, the trees were grown under flood irrigation continuously. DI was adopted in 2018. DI was conducted through two pipes per row; DI tape was placed at a distance of 0.2 m on each side of the tree row. A labyrinth thin-wall DI line with an inner diameter of 16 mm was used with an emitter spacing of 0.3 m and emitter discharge of 2.8 L h^−1^ in a system operating pressure of 0.1 MPa. Based on the traditional flood irrigation amounts (1,100 mm), DI was conducted at 40% (low water volume, LW), 60% (middle water volume, MW), and 80% (high water volume, HW) of the flood irrigation amounts. A total of three treatments with three replicates were conducted in 9 plots. Each plot had an area of 48 m2 (8 m × 6 m), and a separate water gauge was installed on the pipeline to monitor and control the irrigation water volume. DI was conducted 12 times with the same irrigation rate during the growth period.

### 2.2 Sample collection and measurement of soil properties

Soil samples were collected every 20 cm within 0–60 cm below the surface, at horizontal distances of 20 cm from the jujube trunk. A multi-point sampling method was used to collect mixed soil samples for each treatment at three random points in the field, and each treatment was repeated three times. The soil samples were divided into two parts, one for the determination of the soil properties, and another was treated with liquid nitrogen and then cryopreserved at −80°C for bacterial community analyses. For soil properties measurements, soil moisture content was determined by the oven drying method. The concentration of available phosphorus (AP) in the soil was measured by UV–visible spectrophotometer (Madam, UV-1200, China), available nitrogen (AN) was measured by Continuous Flow Analyzer (Seal Analytical, AA3-HR, Germany), Available potassium (AK) was measured by flame photometer (Aucy, FP6450, China), Alkaline phosphatase, Catalase, Polyphenol oxidase, and Urease were measured using the sodium benzoate colorimetric method, UV absorption method, pyrogallol colorimetric method, Indophenol blue colorimetric method, and enzyme-linked analyzer (Tech, Infinite F50, Switzerland) at the wavelength of 450, 240, 450, and 630 nm. Another part of the fresh soil samples was treated with liquid nitrogen for rapid cooling and then cryopreserved at-80°C for determination and analysis of soil microbial communities.

### 2.3 DNA extraction and high-throughput sequencing

The bacterial DNA of soil samples was extracted by the FastDNA Spin Kit for Soil (MP Biomedicals, CA, United States) according to the manufacturer’s instructions. Agarose gel electrophoresis (1.5% concentration) was used to detect the integrity of extracted DNA. Subsequently, the concentration and purity of extracted DNA were measured by NanoDrop 2000 (ThermoFisher, CA, United States). All successfully extracted DNA was stored at −20°C for further application.

The primers 341F (CCTACGGGNGGCWGCAG) and 806R (GGACTACHVGGGTATCTAAT) were used to amplify the V3–V4 regions of the bacterial 16S rRNA gene from each extracted DNA (Berg et al., 2012). PCR reactions were carried out with a 20 μL mixture containing 4 μL of 5 × FastPfu Buffer, 0.4 μL of FastPfu Polymerase, 0.8 μL of each primer (5 μM), 2 μL of 2.5 mM dNTPs, and 10 ng of template DNA. Thermal cycling consisted of initial denaturation at 95°C for 30 s, followed by 25 cycles of denaturation at 95°C for 5 s, annealing at 55°C for 30 s, and elongation at 72°C for 30 s, and a final extension at 72°C for 6 min. The AxyPrep DNA Gel Extraction Kit (Axygen Biosciences, CA, United States) was applied to purify the PCR products, and the concentrations of purified PCR products for each sample were measured by Qubit®3.0 (Life Invitrogen). Then, purified PCR products of each sample were mixed equally, and the sequencing libraries were constructed by the TruSeq Nano DNA LT Library Prep Kit (Illumina, United States). QuantiFluor dsDNA system (Promega, United States) and Agilent Bioanalyzer 2100 system (Agilent, United States) were applied to assess the quality of libraries. Finally, these libraries were sequenced in the Illumina Novaseq6000 platform with the 250 bp paired-end strategy at BIOZERON Biotech. Co., Ltd. in Shanghai, China.

### 2.4 Sequence data processing

The sequenced reads were assigned to each sample based on their unique barcode combined with the end of the reverse primer and truncated by cutting off the barcode sequences. Quality control of sequenced reads was performed based on the following standards: (i) average Phred scores higher than 20, (ii) no ambiguous bases, (iii) homopolymer runs lower than 8, (iv) no mismatches in the primers, and (v) read length longer than 250 bp (Bokulich et al., 2013). Then, the DADA2 plugin unit in the QIIME2 program was run to assemble the paired reads, eliminate the chimera, and cluster clean data into amplicon sequence variants (ASVs; Bokulich et al., 2018). Each ASV was appointed to a taxonomy based on the SILVA database (Release 138; Yilmaz et al., 2014). Finally, singletons (the number of a specific ASV was one) were abandoned and the remained data was normalized using the lowest read number among all samples.

### 2.5 Statistical analysis

All statistical analyses were accomplished by R v4.1.0. Two alpha diversity indices, Chao1 (richness) and Shannon (diversity), of all samples, were calculated (“vegan” package). Differences in available nutrient levels, soil enzyme activities, and alpha diversity of soil bacterial communities between different growth stages (Student’s *t*-test), soil depths (Tukey’s honest significant difference (HSD) test), and water volume of DI (Tukey’s HSD test), were compared. In addition, the effects of growth stage, soil depths, and water volume of DI on soil bacterial communities were assessed by the principal coordinate analysis (PCoA) and Adonis test based on the Bray-Curtis distance (“vegan” and “ape” packages). Student’s *t*-test and Tukey’s HSD test were also executed to identify bacterial phyla and genera with different relative abundance between different growth stages or the same growth season with different water volumes of DI, respectively. Canonical correspondence analysis (CCA) was conducted to determine the contribution of available nutrient levels and soil enzyme activities to the shift of soil bacterial communities (“vegan” package).

To further clarify the community assembly processes, Stegen et al. (2015) developed the null modeling method. The process governing bacterioplankton community assembly was identified based on the beta Nearest taxon index (beta NTI) and Raup-Crick metric (RC). We estimated the relative influence of variable selection or homogeneous selection as the fraction of its comparisons with βNTI > +2 or βNTI < −2, respectively. Subsequently, we divided the remaining pairwise comparisons into |βNTI| ≤ 2 by using the taxonomic diversity metric RC, which ranges from −1 to 1, with values near −1 (−0.95 to −1) indicating homogenizing dispersal (that is, mass effect), values near 1 (0.95 to 1) indicating dispersal limitation and other values (−0.95 to 0.95) indicating drift. During this calculation process, beta mean taxon distance (betaMNTD) between different soil bacterial communities was obtained to assess the phylogenic variation. Linear regression (“lm” function) was applied to evaluate the associations between soil properties with betaMNTD for identifying the potential key factors regulating the bacterial community assembly.

## 3 Results

### 3.1 Variations in soil properties and jujube yield

**Figure 1:**
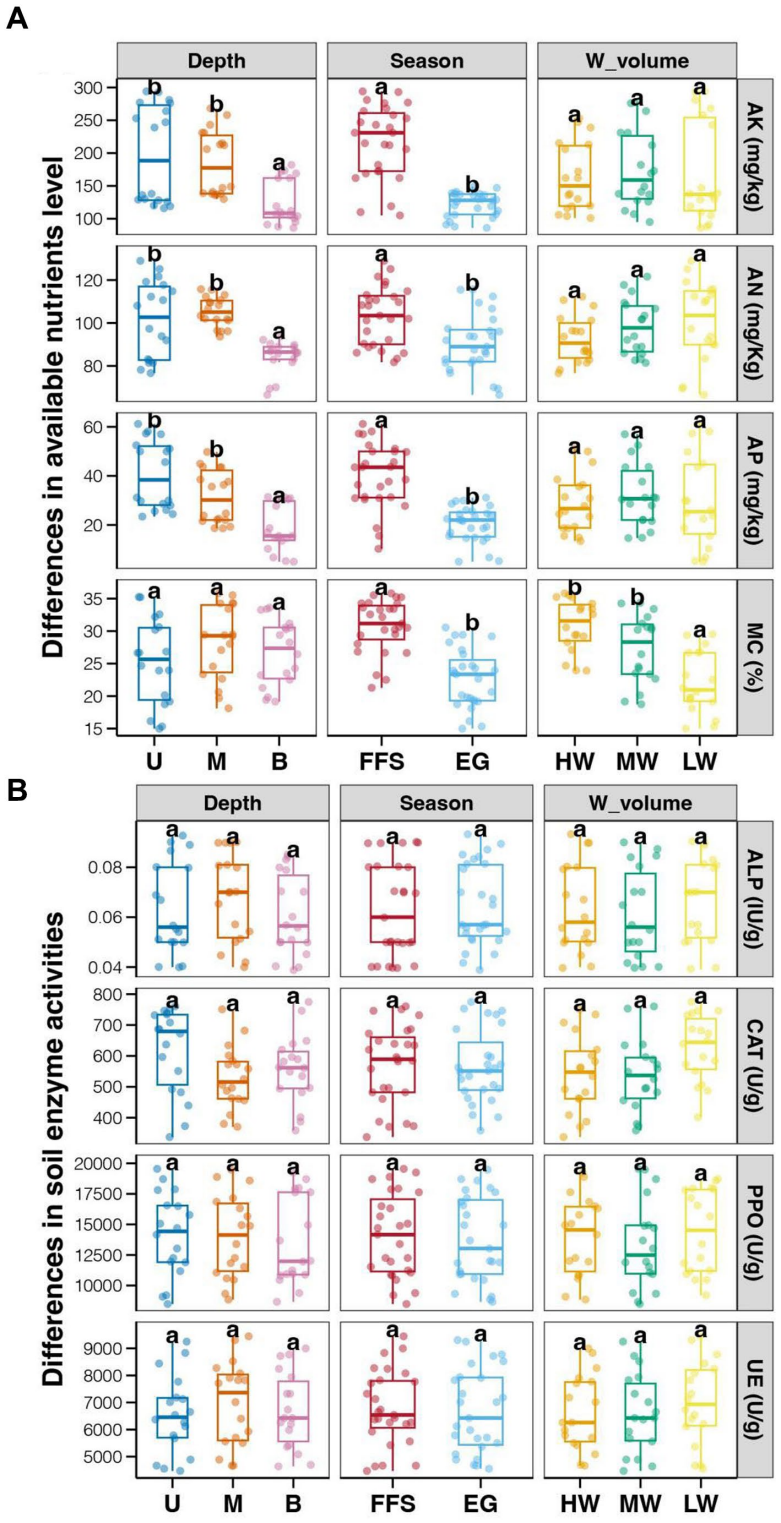
Effects of soil depths, jujube growth stages, and water volume in DI on soil properties. **(A)** available nutrients level; **(B)** soil enzyme activities. Different lowercase letters above each box in the same sub-figure represent significant differences among different samples (Tukey’s HSD test, *p* < 0.05). FFS, flowering and fruit set; EG, end of growth; U, upper; M, middle; B, bottom; HW, high water volume; MW, middle water volume; LW, low water volume.

**Figure 2:**
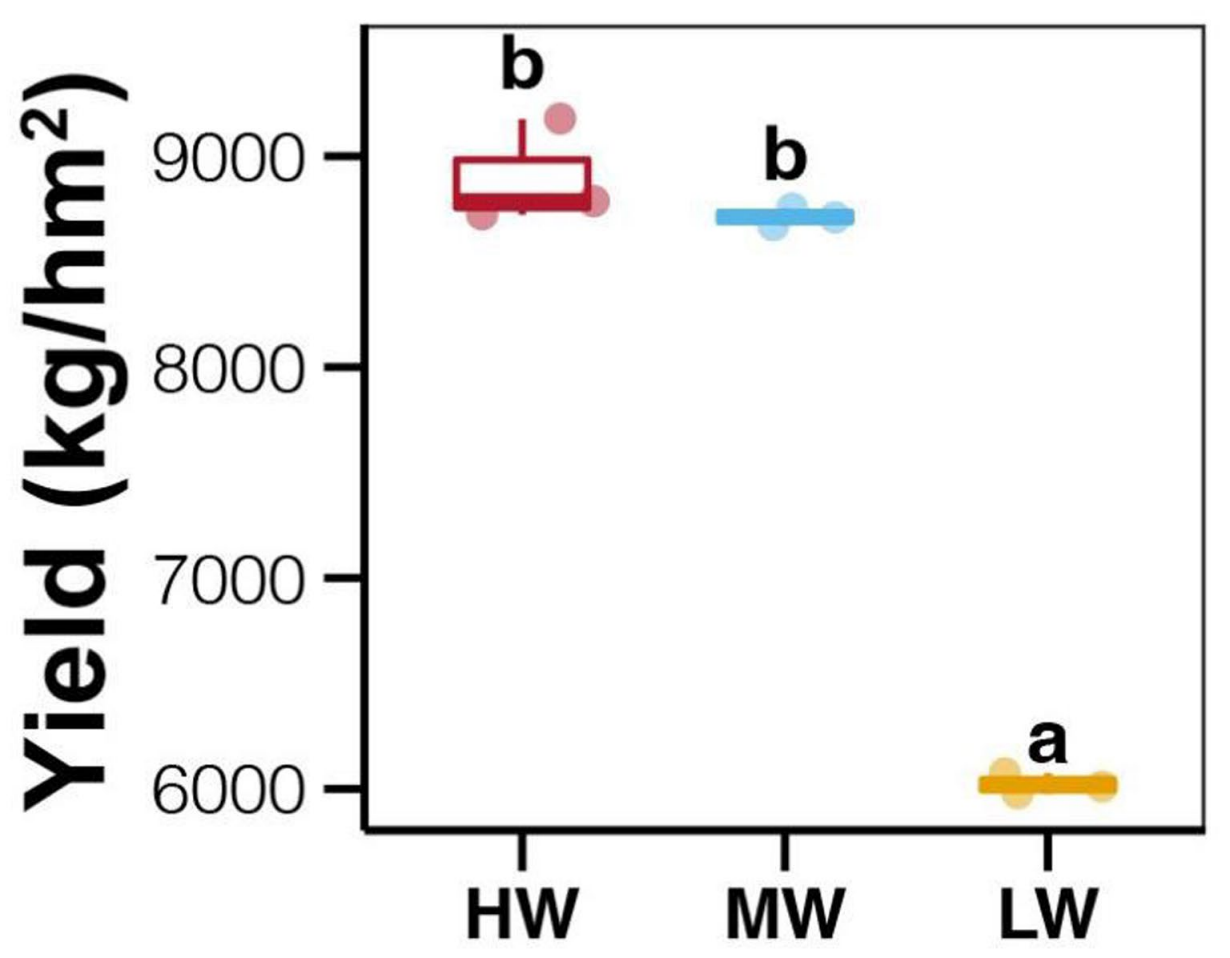
Differences in jujube yields among fields with different water volumes in DI. Different lowercase letters above each box in the same sub-figure represent significant differences among different samples (Tukey’s HSD test, *p* < 0.05).

The average enzyme activities showed relative higher in soil with LW compared to those in MW and HW groups, however, the differences among different water volume groups were not significant (Tukey’s HSD test, *p* > 0.05; Figure 1A). In addition, no significant differences in soil enzyme activities were also observed among different soil depths and jujube growth stages (Tukey’s HSD test, *p* > 0.05; Figure 1A). In contrast, significantly lower ANL (AK, AN, and AP) and MC were found in soils at the EG stage compared to those at the FFS stage (Tukey’s HSD test, *p* < 0.05; Figure 1B). Significantly lower ANL were also revealed in bottom soils compared to upper and middle soils (Tukey’s HSD test, *p* < 0.05; Figure 1B), while MC among different soil depths did not significantly vary (Tukey’s HSD test, *p* > 0.05; Figure 1B). Moreover, the ANL among soils with different water volumes was not significantly different (Tukey’s HSD test, *p* > 0.05; Figure 1B), but the MC was significantly lower in the LW group compared to MW and HW groups (Tukey’s HSD test, *p* < 0.05; Figure 1B). More importantly, we found significantly higher jujube yield in HW and MW fields compared to those in LW fields (Tukey’s HSD test, *p* < 0.05; Figure 2).

### 3.2 Variations in soil bacterial communities

**Figure 3:**
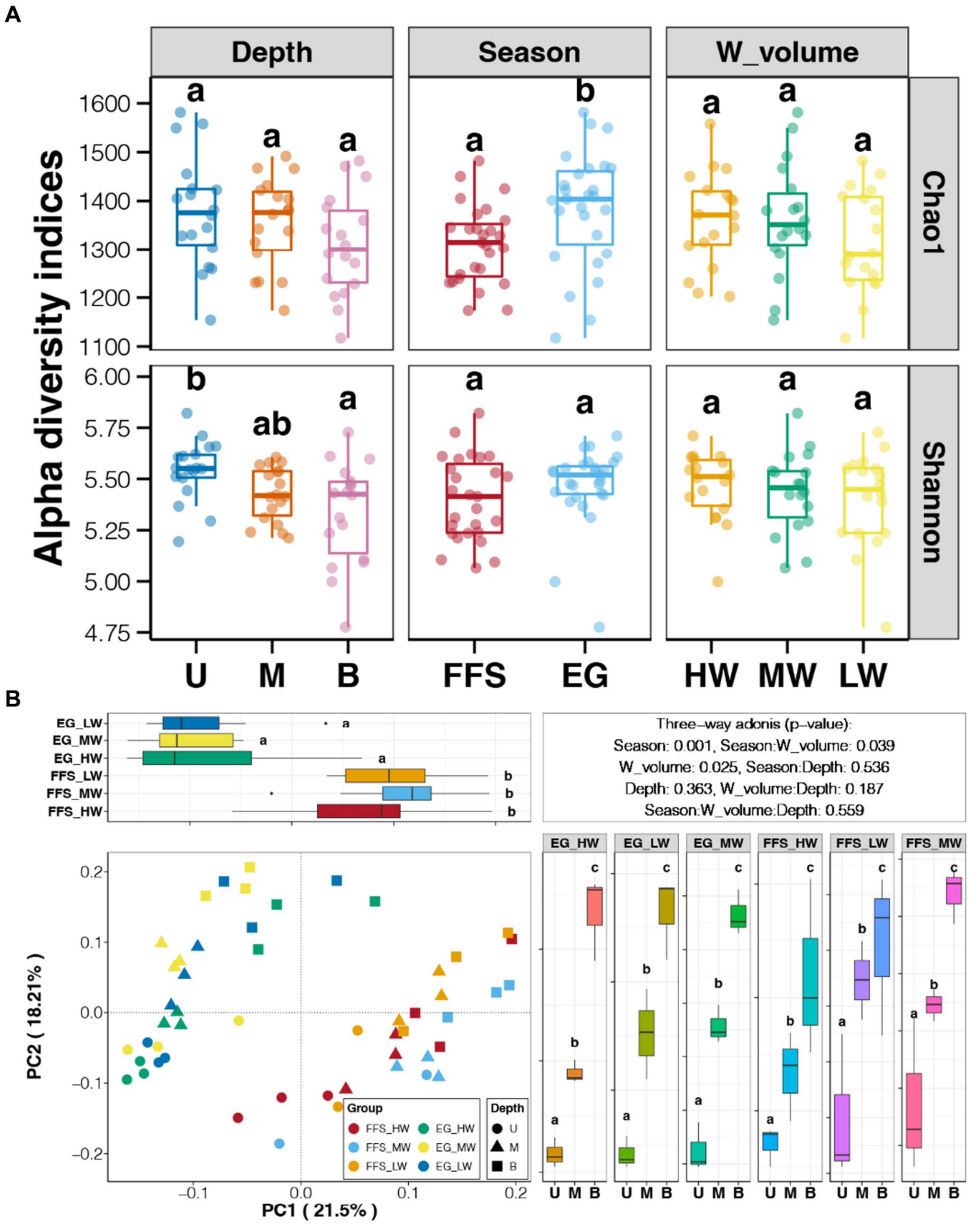
**(A)** Variations in alpha diversity indices among soil bacterial communities with different growth seasons, irrigation methods, and soil depths, respectively. **(B)** Principal coordinate analysis (PCoA) and adonis test of shrimp gut microbiota from different aquaculture seasons based on Bray-Curtis distance. Different lowercase letters above each box in the same sub-figure represent significant differences among different samples (Tukey’s HSD test, *p* < 0.05). FFS, flowering and fruit setting; EG, end of growth; U, upper; M, middle; B, bottom; HW, high water volume; MW, middle water volume; LW, low water volume.

According to the comparisons of alpha diversity indices of soil bacterial communities among different groups, we found significantly higher richness in the EG stage compared to the FFS stage and an obviously decreased trend of diversity alongside the soil depth (Figure 3A). However, the water volume of DI showed no significant impact on the alpha diversity of soil bacterial communities (Tukey’s HSD test, *p* > 0.05; Figure 3A). As shown in PCoA, soil bacterial communities from the different jujube growth stages are separated by the PC1 axis, which explains 21.5% of the total variation (Figure 3B). In contrast, no significant difference in bacterial communities was revealed among soils with different water volumes but the same growth stage (Tukey’s HSD test, *p* > 0.05; Figure 3B). In addition, significant clusters according to the soil depths were observed along the PC2 axis (Tukey’s HSD test, *p* < 0.05), which explained 18.21% of the total variation (Figure 3B). Moreover, three-way adonis indicated significant effects of jujube growth stage and water volume of DI on soil bacterial communities (*p* < 0.05; Figure 3B).

**Figure 4:**
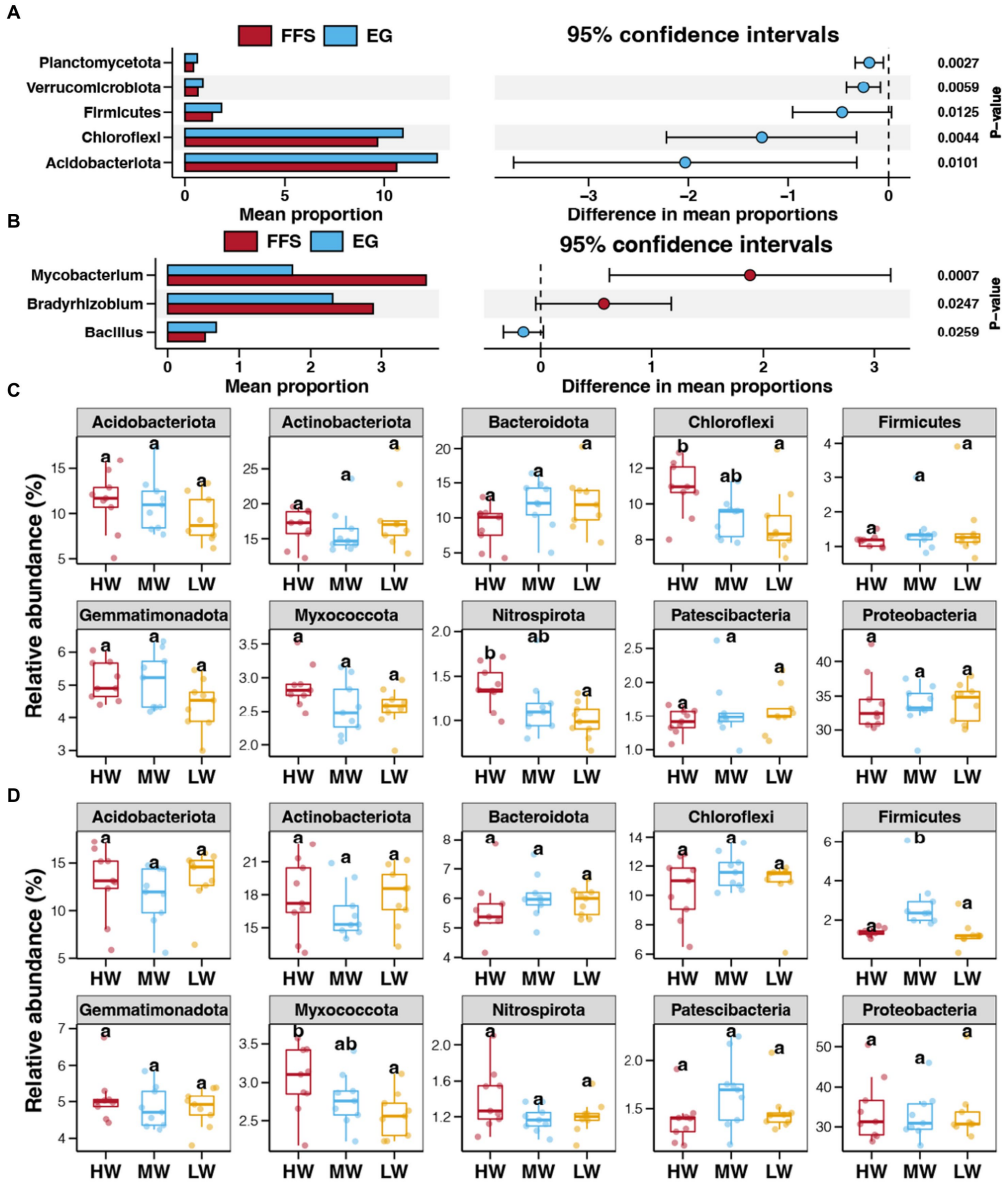
Differences in the relative abundances of bacterial phyla **(A)** and genera **(B)** between soils from the FFS and EG stages. Differences in the relative abundance of dominant bacterial phyla in soils with different water volumes of DI at the FFS **(C)** and EG **(D)** stages, respectively. Different lowercase letters above each box in the same sub-figure represent significant differences among different samples (Tukey’s HSD test, *p* < 0.05).

Proteobacteria was the dominant bacterial phyla in all investigated soils, followed by Actinobacteriota, Acidobacteriota, Chloroflexi, Bateroidota, and Gemmatimonadota (Supplementary Figure S1). At the genus level, the most dominant bacteria were *Vibrionimonas*, followed by *Mycobacterium*, *Bradyrhizobium*, *Burkholderia-Caballeronia-Paraburkholderia*, *MND1*, *Sphingomonas* (Supplementary Figure S2). Based on the results of bacterial diversity, we first compared the bacterial abundances between soils from the FFS and EG stages. In contrast to the FFS stage, the relative abundances of Acidobacteriota, Chloroflexi, Firmicutes, Verrucomicrobiota, and Planctomycetota significantly increased in the EG stage (Student’s *t*-test, *p* < 0.05; Figure 4A). For bacterial genera, *Bacillus* was significantly enriched in the EG stage, while *Mycobacterium* and *Bradyrhizobium* were more abundant in the FFS stage (Figure 4B). We further investigated the effects of the water volume of DI on bacterial abundances at each of the single jujube growth stages. A higher water volume of DI decreased the relative abundance of Chloroflexi and Nitrospirota in soils from the FFS stage (Figure 4C). At the EG stage, a higher water volume of DI decreased the relative abundance of Myxococcota, and more abundant Firmicutes were observed in the MW group compared to HW and LW groups (Figure 4D).

### 3.3 Variations in assembly processes of soil bacterial communities

**Figure 5:**
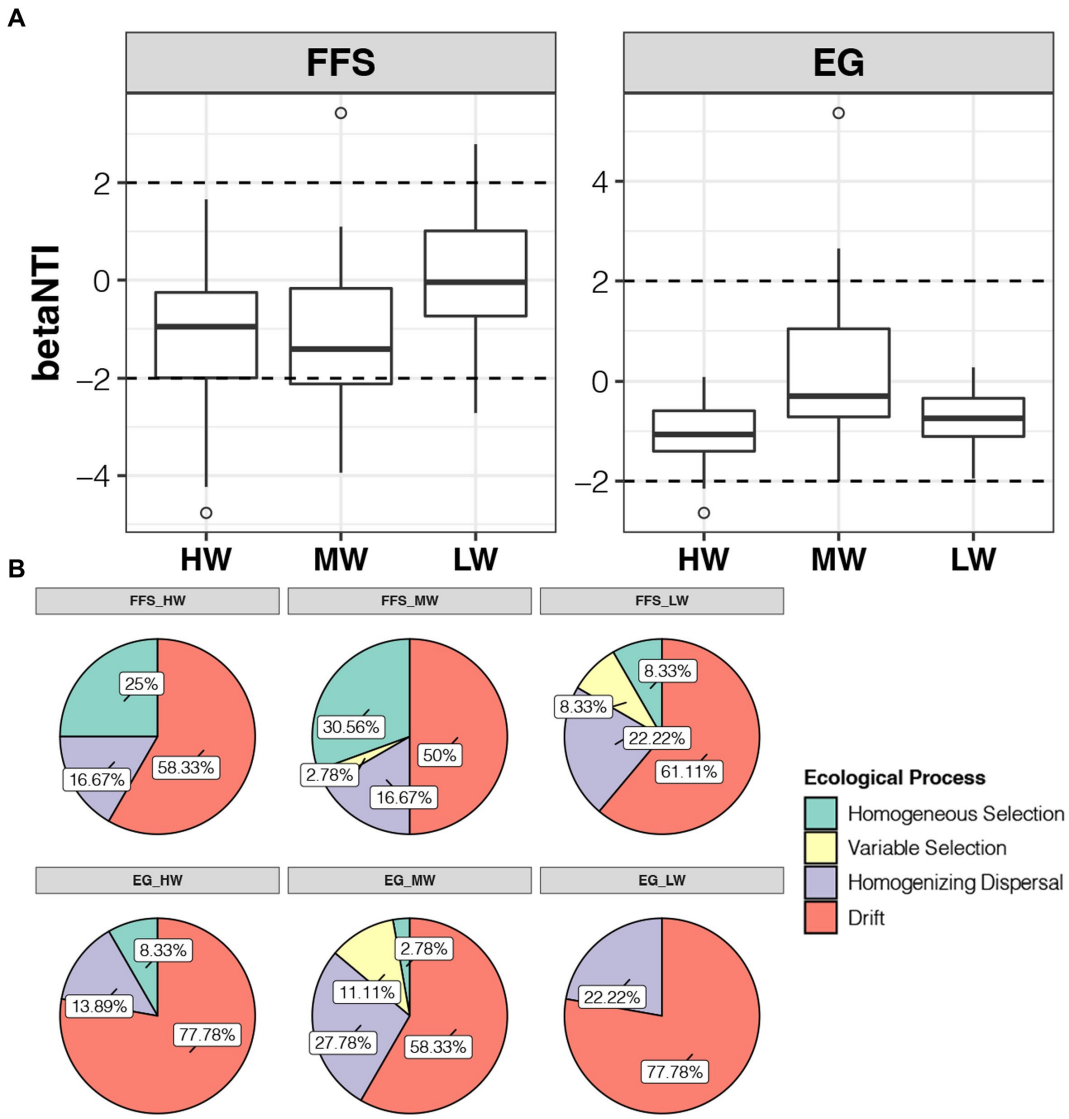
**(A)** Differences in betaNTI of soil bacterial communities under different irrigation treatments at the FFS and EG stages, respectively. **(B)** Ratios of different ecological processes for the assembly of soil bacterial communities under different irrigation treatments at the FFS and EG stages, respectively.

The median of betaNTI for all groups was between-2 to 2, indicating stochastic processes dominate the assembly of soil bacterial communities no matter which jujube growth stages or water volume of DI (Figure 5A). At the FFS stage, the median of betaNTI in the LW group was closer to 0 than those in the HW and MW groups, indicating higher water volume of DI decreased the contribution of stochastic processes for the assembly of soil bacterial communities (Figure 5A). These decreases were reflected in the lower rate of homogenizing dispersal and drift in samples of the HW (16.67% and 58.33%) and MW (16.67% and 50%) groups compared to the LW (22.22% and 61.11%) group at the FFS stage (Figure 5B). At the EG stage, although the median of betaNTI in the MW group was closest to 0, all values in the LW group were between-2 to 2, indicating the assembly of soil bacterial communities in the LW group was all governed by the stochastic processes (Figure 5A). The decreases of stochastic processes in HW and MW groups compared to the LW group at the EG stage were reflected in the lower rate of homogenizing dispersal (13.89% vs. 22.22%) and drift (58.33% vs. 77.78%), respectively (Figure 5B). For deterministic community assembly, it was more important in the MW (38.34% and 13.89%) group than HW (25% and 8.33%) and LW (16.66% and 22.22%) groups at both FFS and EG stages (Figure 5B). Among them, homogeneous selection and variable selection were contributed more at the FFS and EG stages, respectively (Figure 5B). Taken together, our results suggested lower and middle water volumes of DI induced higher contribution of stochastic and deterministic processes, respectively, for the assembly of soil bacterial communities.

### 3.4 Correlations between soil properties and soil bacterial communities

**Figure 6:**
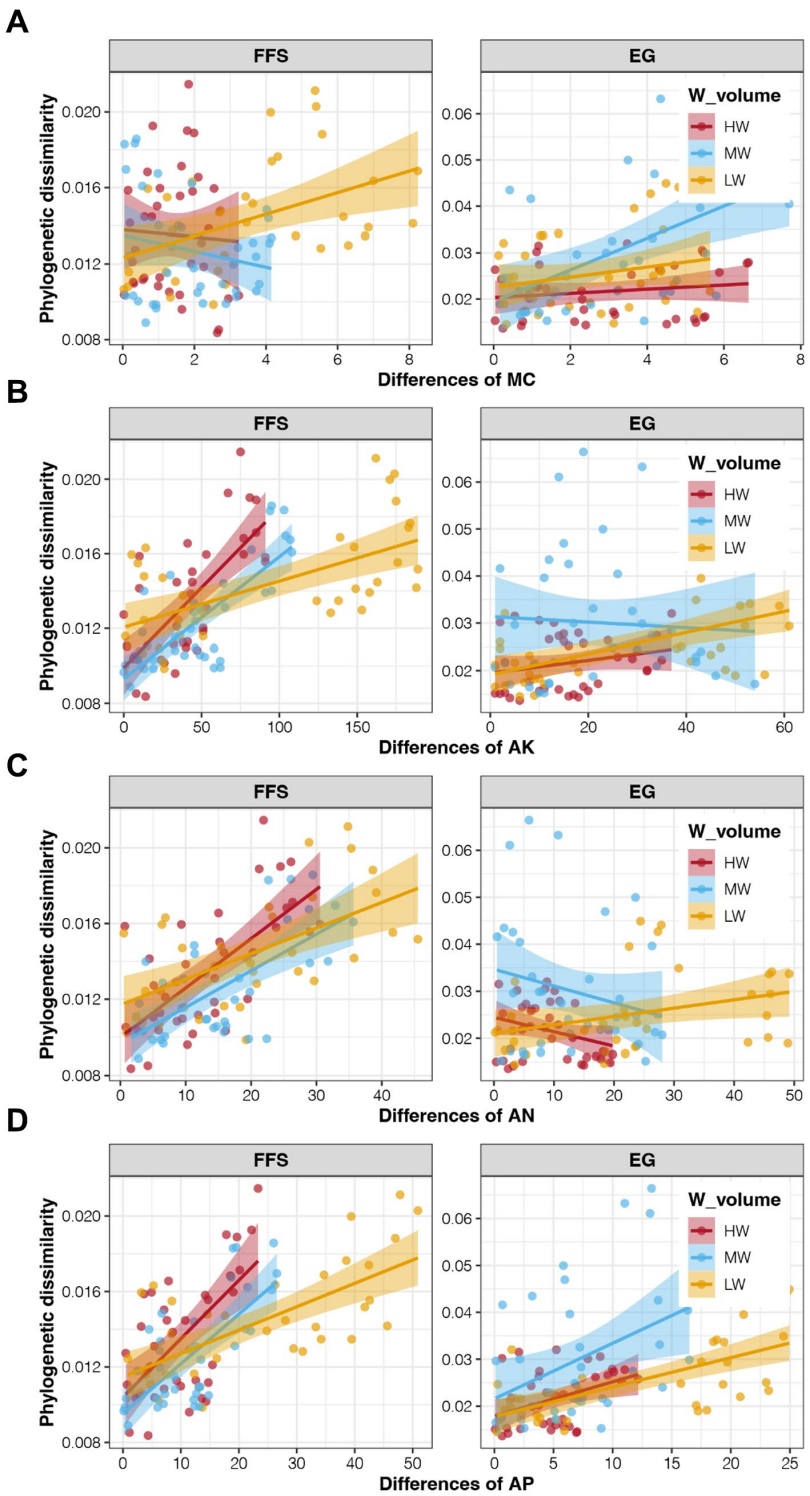
Correlations between the soil properties and betaMNTD of soil bacterial communities in different irrigation treatments and jujube growth stages. **(A)** MC, **(B)** AK, **(C)** AN, **(D)** AP.

CCA revealed the associations between soil properties and soil bacterial communities, and the results are shown in Supplementary Figure S3. The first two PCs explained a total of 53.85% variations of bacterial communities, indicating obvious effects of soil properties. More importantly, only three ANL indices and MC showed significant correlations with the soil bacterial communities, but not for all four soil enzyme activities (*p* < 0.05; Supplemenatry Table S1). We further investigated the associations between these four potential key soil properties with the assembly of soil bacterial communities. At the FFS stage, a significant correlation was only obtained between the MC and betaMNTD of bacterial communities in LW soils (Figure 6A; Supplementary Table S2), indicating obvious effects of water volume of DI on soil bacterial community assembly. In addition, the content of AN, AP, and AK were all significantly correlated to the betaMNTD of soil bacterial communities in all three irrigation treatments at the FFS stage, which were all strongest in the MW group (*p* < 0.05; Figures 6B–D; Supplementary Table S2). At the EG stage, significant correlations between the MC and AP with the betaMNTD of soil bacterial communities were found in the MW group (Figures 6A–D; Supplementary Table S2). These results were consistent with the highest contribution of deterministic processes for bacterial community assembly in soil with middle water volume.

## 4 Discussion

DI is a highly efficient and effective method for water and nutrient delivery in agriculture. Its benefits in water conservation, crop productivity, and environmental sustainability make it a valuable tool for farmers worldwide (Pérez-Blanco et al., 2020). The usage of DI in agriculture has been reported to have the ability to improve soil properties and change soil microbial communities (Akhtar et al., 2018; Jat et al., 2018; Shen et al., 2018). In the present study, the effects of growth stage, soil depth, and water volume of DI on soil properties and bacterial communities of jujube fields were investigated. Significant impacts of water volume of DI on soil MC and jujube yield were observed. An important purpose of designing the DI technology is to achieve maximum crop yield by minimizing water consumption (Pretty et al., 2010). The specific optimal amount of water for drip irrigation to maximize crop yield may vary depending on specific crop and environmental conditions (Çetin and Akalp, 2019). Some studies have reported that optimal irrigation levels improved crop growth and yield. According to a review of drip irrigation in China, when the drip irrigation amount is more (100%–120%), drip irrigation significantly increases crop yields by 28.92%, 14.55%, 8.03%, 2.32%, and 5.17% relative to other irrigation methods (Yang et al., 2023). Another study conducted in China focused on maize growth and yield under DI and found that optimal irrigation levels improved maize growth, yield, and water use efficiency (Liu et al., 2022). However, most of them did not mention the specific optimal water amount (Li et al., 2021a; Wang et al., 2022). According to our results, a middle water amount (660 mm) of DI was necessary to achieve high jujube yields.

In contrast to MC and jujube yield, the effects of the water volume of DI on ANL and bacterial communities were weaker than in jujube growth stages. This phenomenon was reasonable due to the significant variations of soil during different crop growth stages (Shen et al., 2018). Soil bacterial communities have been found to vary across different growth stages of the various plants, with changes observed in the rhizosphere and bulk soil (Navarro-Noya et al., 2022; Xie et al., 2023). The changes of soil bacterial communities over the crop cycle could be influenced by factors such as water content and nutrient levels (Romero-Salas et al., 2021). This is consistent with our results of significant correlations between soil bacterial community structure and ANL and MC covering samples from both FFS and EG stages. Moreover, the relative abundance of bacterial phyla and species detected across all plant growth stages can vary, with bacterial communities in the bulk and rhizosphere soils showing differences across different developmental stages (Ajilogba et al., 2022). Some studies related to potential disease and jujube tree health mentioned the bacterial genus of *Mycobacterium*, which was more abundant in the FFS stage, however, there was no direct evidence of the effects of *Mycobacterium* on jujube health (Kwon et al., 2019; Ran et al., 2022). There was one study that investigated the effect of *Bradyrhizobium*, another bacterial genus that enriched in the FFS stage, on jujube growth, and suggested it as a potential biofertilizer for jujube trees (Bitire et al., 2022). In addition, another study mentioned *Bradyrhizobium* could enhance the drought tolerance of the jujube tree (Zhang et al., 2020a). Moreover, previous studies provided evidence that *Bacillus*, the bacterial genus enriched in the EG stage, has a potential antagonistic effect on various plant pathogenic fungi (Chen et al., 2019), controls fungal diseases (Kwon et al., 2021), and increases production (Zhou et al., 2021) of jujube fruit.

We further realized the changes in main soil bacteria at each single growth stage. Chloroflexi was observed to be negatively correlated with soil MC in the Tibetan Plateau and Antarctica (Kim et al., 2015; Li et al., 2022), however, higher MC was associated with a higher abundance of Chloroflexi in samples at the FFS stage of this study. Meanwhile, soil MC was determined to be a primary driver of Nitrospira (Wang et al., 2023), which was also found more abundant in soils with higher MC at the FFS stage in the present study, and its abundance was positively correlated with annual rainfall (Chisholm et al., 2023). Moreover, one study about coastal saline soil reported that Myxococcota was negatively correlated with soil MC (Tian et al., 2023), in contrast, which was positively correlated to MC in soils at the EG stage of our study. The inconsistent results may be due to the different environmental systems that previous studies focused on compared to this work. Therefore, more research is needed to determine the relationship between soil bacteria and MC in different soil types and environments.

Finally, assembly mechanisms of soil bacterial community and their association to soil properties under different water volumes of DI were explored. Our results revealed an increase in the contribution of deterministic processes on soil bacterial communities for samples from the jujube fields receiving the middle water volume of DI. This is probably because this DI strategy changed some soil properties, which could act as the deterministic filtering factor to select certain bacteria in soils (Jiao and Lu, 2020). The strongest correlations between the ANLs and soil bacterial communities obtained in our results confirmed this speculation. It is possible that available soil nutrients have an effect on certain microorganisms by significantly altering the crop growth efficiency (Ramirez et al., 2010; Zhao et al., 2019). In our study, we observed significant enrichment of Firmicutes in soils with middle water amount. A study by Xia et al. (2023) found that lower soil pH led to higher Firmicutes abundance, which was positively correlated with rice yield under milk vetch rotation. Another study by Li et al. (2021b) found that Firmicutes were significantly and positively correlated with the yield of winter wheat, which could be regulated by soil organic carbon. Overall, these findings suggest that there may be a correlation between soil Firmicutes and crop yield, although the relationship may be influenced by other factors such as soil pH, organic carbon, and water content.

## 5 Conclusion

In summary, the results of this study provided evidence that the water volume of DI could change the soil bacterial communities and then regulate the resultant jujube yields. The water volume of DI demonstrated the MC and jujube yields of studied agriculture systems, while jujube growth stages were more important for soil ANL and bacterial communities. The genera of *Mycobacterium* and *Bradyrhizobium* were more abundant in the FFS stage while *Bacillus* was enriched in the EG stage. When the seasonal effects were restricted, Chloroflexi, NItrospirota, and Myxococcota showed a positive correlation with soil water amount. More importantly, the middle water amount of DI increased the deterministic assembly of the soil bacterial community. This increase correlated to the variations of ANLs in soils and resulted in the enrichment of Firmicutes and increased the jujube yields. Based on our results, we could deduce the middle water amount (660 mm) as the optimal condition for DI during jujube cultivation.

## Data availability statement

The original contributions presented in the study are included in the article/Supplementary material, further inquiries can be directed to the corresponding author.

## Author contributions

ZL: Data curation, Methodology, Software, Writing – original draft, Writing – review & editing. YY: Data curation, Formal analysis, Writing – review & editing. JL: Data curation, Writing – review & editing. WJ: Data curation, Writing – review & editing. YG: Conceptualization, Investigation, Validation, Writing – review & editing.
