# Supplementary material for: Effects of water volume of drip irrigation on soil bacterial communities and its association with soil properties in jujube cultivation
Source: Front Microbiol. 2024 Jan 19;14:1321993. doi: 10.3389/fmicb.2023.1321993 (PMC10836404; doi:10.3389/fmicb.2023.1321993)
Supplement: Supplementary file 1 [file Data_Sheet_1.docx]

**Supporting information for**

#### **Effects of water volume of drip irrigation on soil bacterial communities and its association with soil properties in jujube cultivation**

**Table S1.** Results of CCA for soil properties for bacterial communities in jujube fields with different water volumes of DI.

| **Soil properties** | **R^2^** | ***p-value*** |
| --- | --- | --- |
| ALP | 0.025 | 0.523 |
| CAT | 0.044 | 0.288 |
| PPO | 0.040 | 0.34 |
| UE | 0.012 | 0.747 |
| AP | 0.711 | 0.001 |
| AK | 0.660 | 0.001 |
| AN | 0.275 | 0.002 |
| MC | 0.473 | 0.001 |

**Table S2.** Correlation results between the soil properties with betaMNTD between bacterial communities among different treatments

| **Soil properties** | **Stage** | **Group** | **R^2^** | ***p-value*** |
| --- | --- | --- | --- | --- |
| MC | FFS | HW | -0.027 | 0.771 |
|  |  | MW | 0.015 | 0.222 |
|  |  | LW | 0.193 | 0.004** |
|  | EG | HW | -0.004 | 0.363 |
|  |  | MW | 0.233 | 0.002** |
|  |  | LW | 0.021 | 0.193 |
| AK | FFS | HW | 0.495 | 1.01E-06*** |
|  |  | MW | 0.572 | 5.68E-08*** |
|  |  | LW | 0.362 | 6.25E-05*** |
|  | EG | HW | 0.028 | 0.167 |
|  |  | MW | -0.026 | 0.735 |
|  |  | LW | 0.305 | 2.86E-04*** |
| AN | FFS | HW | 0.448 | 4.78E-06*** |
|  |  | MW | 0.425 | 1.01E-05*** |
|  |  | LW | 0.333 | 1.35E-04*** |
|  | EG | HW | 0.055 | 0.090 |
|  |  | MW | 0.020 | 0.197 |
|  |  | LW | 0.085 | 0.047* |
| AP | FFS | HW | 0.365 | 5.77E-05*** |
|  |  | MW | 0.495 | 1.02E-06*** |
|  |  | LW | 0.459 | 3.44E-06*** |
|  | EG | HW | 0.140 | 0.014* |
|  |  | MW | 0.132 | 0.017* |
|  |  | LW | 0.439 | 6.41E-06*** |

**Figure S1.** Relation abundances of top 10 bacterial phyla in studied soil samples.

**Figure S2.** Relation abundances of top 10 bacterial genera in studied soil samples.

**Figure S3.** CCA for correlations between soil properties and bacterial communities.
